# Ultra-Stable, Conductive, and Porous P-Phenylenediamine-Aldehyde-Ferrocene Micro/Nano Polymer Spheres for High-Performance Supercapacitors with Positive Electrodes

**DOI:** 10.3390/polym17141964

**Published:** 2025-07-17

**Authors:** Xin Wang, Qingning Li, Zhiruo Bian, Da Wang, Cong Liu, Zhaoxu Yu, Xuewen Li, Qijun Li

**Affiliations:** 1 School of Materials Science and Chemical Engineering, Harbin University of Science and Technology, Harbin 150080, China; lqn20010702@163.com (Q.L.); bianzr123@163.com (Z.B.); wagnda@163.com (D.W.); l17860391809@163.com (C.L.); ttkong1998@163.com (Z.Y.); 2 Key Laboratory for Light-Weight Materials, Nanjing Tech University, Nanjing 211816, China; lixw@njtech.edu.cn; 3 School of Mechanical Engineering, Institute of Technology for Carbon Neutralization, Yangzhou University, Yangzhou 225009, China

**Keywords:** poly-Schiff, electrode materials, supercapacitor, electrochemical performance

## Abstract

Supercapacitors, with their remarkable attributes such as including a high power density, an extended cycle life, and inherent safety, have emerged as a major research area for electrochemical energy storage. In this paper, phenylenediamine and glyoxal were used as raw material to prepare p-phenylenediamine glyoxal (PGo) with one single step. p-phenylenediamine glyoxal-ferrocene (PGo-Fc_x_, x = 1, 0.3, 0.2, 0.1) composites were synthesized based on a poly-Schiff base. FTIR and XRD results indicated that ferrocene doping preserves the intrinsic PGo framework while inducing grain refinement, as evidenced by the narrowing of the XRD diffraction peaks. SEM observations further revealed distinct morphological evolution. CV (cyclic voltammetry), EIS (electrochemical impedance spectroscopy), and GCD (galvanostatic charge–discharge) were conducted on PGo-Fc_x_ in order to examine its electrochemical performance. The PGo-Fc_0.3_ in PGo-Fc_x_ electrode material had a specific capacitance of 59.6 F/g at a current density of 0.5 A/g and 36 F/g at a current density of 10 A/g. Notably, even after undergoing 5000 charging–discharging cycles at 10 A/g, the material retained 76.2% of its specific capacitance compared to the initial cycle. Therefore, taking conductive polymers and metal oxide materials for modification can improve the stability and electrochemical performance of supercapacitors.

## 1. Introduction

Energy is the cornerstone of modern human society. Before the 21st century, the most widely used energy sources were fossil fuel, such as coal, oil, and natural gas. However, with the development of the economy and society, green energy, new energy, and environment-friendly energy have received more and more attention. At the same time, many countries have put forward ideas such as “carbon peak” and “carbon neutrality”, which have encouraged the development of new energy [1,2,3]. To broaden the utilization of newer and greener energy sources, more and more attention is being paid to wind power, nuclear power, solar power, etc., but the conversion and storage of these energy sources have become a key issue [4,5]. Currently, the growing emphasis on research on batteries and supercapacitors stems from their increasing deployment in electric vehicles, smart devices, industrial operations, and energy infrastructure, which is coupled with rapid technological progress [6,7]. It is well known that, compared with rechargeable batteries, supercapacitors possess a higher power density and charging–discharging rate for electrostatic adsorption and redox reactions on the surface of the electrode material rather than the embedding and de-embedding of ions [8]. The electrochemical characteristics of the electrode materials serve as the determining factors for the performance of supercapacitors, which makes electrode material research the central focus of technological advancements in this field.

Supercapacitors, also known as “supercapacitor-batteries”, are power energy storage devices that occupy a space between traditional capacitors and batteries. Supercapacitors and lithium-ion batteries represent two types of more advanced energy storage devices. Lithium-ion batteries, due to their higher energy density, have been widely used in fields such as in electronic equipment and transportation. However, lithium-ion batteries also have certain problems, such as the slow transmission of electrons and ions due to a variety of resistive losses, that the battery will produce a large amount of heat in high-power operation, and that the battery’s dendritic phenomenon is likely to lead to safety issues. Compared with batteries, supercapacitors have the advantages of a high power density, fast charging and discharging, a long cycle life, and strong environmental adaptability [9]. Wang et al. [10] synthesized carbon nanotube film-supported Bi_2_O_3_ electrodes with a remarkable capacity of 1.90 mAh·cm^−2^ at 1 mA·cm^−2^, and CNT@NiCo_2_O_4_//CNT@Bi_2_O_3_-G provided a maximum energy density of 589.3 μWh·cm^−2^ (98.2 Wh·kg^−1^) at a power density of 0.8 mW·cm^−2^, retaining a capacity of 80.1% after 8000 cycles. Chen et al. [11] prepared an amorphous NiCoMn-OH electrode using a mixed solvent strategy that had a specific capacity of 1124 C·g^−1^, while the specific energy of the supercapacitor-battery NiCoMn-OH//RGO was 42.8 Wh·kg^−1^ at 749 W·kg^−1^. The electrode material was the most important component of the supercapacitor, as it determined the specific capacitance, cycle stability, and rate performance [12]. Activated carbon material has the advantages of low cost, good conductivity, simple preparation, and stable electrochemical properties. However, it has a long diffusion distance and high ion transfer resistance at high current densities, which can lead to sharp decreases with increases in the charging–discharging rate [13,14]. Metal oxides are generally pseudocapacitor supercapacitor electrode materials and have a higher energy density than carbon when they are used as supercapacitor electrode materials [15,16]. Conductive polymers were first discovered by the Japanese scientist Hideki Shirakawa, and since then three major conductive polymers, namely polyaniline (PANI), polypyrrole (PPy), and polythiophene (PTh), have been gradually developed by scientists. These polymers have a highly π–π conjugated polymer backbone and store charge mainly through doping and de-doping [17]. The advantages of conducting polymers are better electrical conductivity, a simple and low-cost preparation process, that their polymer materials are easier to process, and that they have a large specific capacitance [18]. For example, polyaniline has a specific capacitance of 775 F/g and polypyrrole’s is 480 F/g, but the structural stability of these conductive polymers is poor, and their structure will be destroyed or even collapse after many charge–discharge cycles [19,20]. However, PPD has exhibited excellent electrical conductivity, which was a fundamental requirement for its application. Compared to the other conductive polymers mentioned, PPD can achieve higher conductivity levels under appropriate synthesis and doping conditions. In contrast, certain forms of PANI may require more complex doping procedures to reach similar conductivity values, and PPy can sometimes suffer from conductivity degradation over time due to environmental factors.

Therefore, in this paper, a poly-Schiff base with good capacitance and cycling stability was prepared by using phenylenediamine and glyoxal as raw materials, which were doped with different ratios of ferrocene and different ratios of p-phenylenediamine glyoxal-ferrocene (PGo-Fc_x_) using the one-pot method.

## 2. Materials and Methods

### 2.1. Experimental Material

The experimental materials used in this paper are shown in Table 1. P-phenylenediamine is an amine-based compound that was used in the Schiff base reaction. Four different poly-Schiff base materials were prepared using the same amine-based compound and different carbonyl compounds. To remove any potential water, polyvinylidene fluoride (PVDF) was dried in an oven at 80 °C for 6 h before use.

### 2.2. Preparation of Poly-Schiff Base Ferrocene

**Figure 1:**
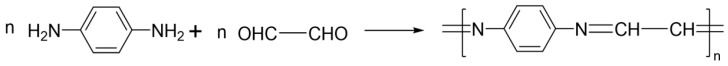
Reaction equation for PGo.

Initially, 2.5 mmol of p-phenylenediamine was dissolved in 30 mL of anhydrous ethanol to form solution A, while 2.5 mmol of a 40% glyoxal solution was dissolved in 10 mL of anhydrous ethanol to create solution B. Separately, 2.5 mmol each of aluminum nitrate nonahydrate, cobalt nitrate hexahydrate, and anhydrous copper nitrate was dissolved in 30 mL of anhydrous ethanol, resulting in solutions C, D, and E, respectively. Next, solution B was gradually and uniformly added to solution A, which led to a color change. Prior to the solution becoming cloudy, solutions C, D, and E were slowly introduced into the mixture of solutions A and B, with continuous magnetic stirring at room temperature for a duration of 6 h. We washed the filter repeatedly 3–5 times with deionized water and anhydrous ethanol, and dried it at 80 °C for 12 h. According to the different materials added, the different molar ratios of p-phenylenediamine glyoxal-ferrocene were named PGo-Fc_1_, PGo-Fc_0.3_, PGo-Fc_0.2_, and PGo-Fc_0.1_, respectively. The reaction equation for PGo is shown in Figure 1.

### 2.3. Preparation of Working Electrode

Nickel foam served as the collector for the working electrode. For active substance coating, a pre-treatment process was carried out to eliminate surface oil and oxide layers. The nickel foam was initially cut into 1 cm × 1 cm square pieces. These pieces were then submerged in acetone for 15 min, which was followed by 5 min of ultrasonic cleaning. Subsequently, they were immersed in a 2 M hydrochloric acid solution and subjected to another 15 min ultrasonic treatment. Finally, the treated foam nickel was repeatedly ultrasonically cleaned 3–5 times with deionized water and absolute ethanol and then vacuum dried in vacuum oven at 80 °C for 12 h. The working electrode was fabricated using a blend of active material, conductive carbon black, and polyvinylidene fluoride (PVDF). These were combined in an onyx mortar at a weight ratio of 8:1:1 and ground thoroughly until a uniform mixture was achieved. N-methyl pyrrolidone (NMP) was gradually added dropwise to the mixture until a viscous, honey-like consistency was acquired. This slurry was then evenly applied onto a pretreated nickel foam substrate. After coating, the sample was vacuum dried at 80 °C for 12 h to remove any residual solvent and ensure proper adhesion of the electrode materials.

### 2.4. Methods for Characterizing Material Structure and Morphology

#### 2.4.1. Fourier Transform Infrared Spectrometer (FT-IR)

Fourier Transform infrared spectrometry (FT-IR) is a technique for qualitatively analyzing the functional groups and chemical bonds of a substance. When infrared radiation passes through a sample, part of the radiation is absorbed by the sample and the other part passes through the sample. The signal generated in the detector is presented in spectral form and represents the molecular “fingerprint” of the sample. Different chemical structures produce a variety of spectral fingerprints, so that compounds can be analyzed and identified. Infrared absorption spectra are caused by molecular vibrations and rotational transitions, and different functional groups have vibrational absorptions at different frequencies, which means that different functional groups have different characteristic peaks in the infrared spectrum. The FT-IR involved in this paper uses radiation source monochromatic Al Kα X-rays with a calibration peak of 284.60 ev for the standard peak of C 1s and a wave number range of 4000–400 cm^−1^.

#### 2.4.2. X-Ray Diffraction (XRD)

X-ray diffraction (XRD) is a technique used to characterize the structure of crystals and their changing laws. The working principle of XRD is that a beam of monochromatic X-rays is incident on the crystal and, due to the fact that crystals are composed of regularly arranged cells and that the distance between these regularly arranged atoms is of the same order of magnitude as the wavelength of the incident X-rays. The X-rays scattered by different atoms interfere with each other and produce strong X-ray diffraction in certain special directions. The orientation and intensity of the diffraction lines in the spatial distribution are closely related to the crystal structure. Diffraction lines from different angles of the crystal surface are collected to obtain the maps of different materials, so as to analyze the physical phase and crystal structure. The parameters of the XRD used in this thesis are a tube voltage of 40 kv, a tube current of 40 mA, a step angle of 0.02°, and a 2θ scanning range between 5° and 80°.

#### 2.4.3. Scanning Electron Microscope (SEM)

Scanning electron microscopy (SEM) is a technical means to observe the surface morphology or cross-section morphology of a material. The working principle of SEM is that an electron gun generates an electron beam which is focused by an electromagnetic lens, and then a scanning coil controls the electron beam to scan the surface of the sample to generate secondary electrons, scattered electrons, reflected electrons, etc., which are converted into electrical signals and finally processed into image information by a detector. The SEM images in this paper were prepared before the test. Specifically, the sample was pasted on the conductive adhesive, then sprayed with gold, and finally scanned.

### 2.5. Electrochemical Performance Testing

The electrode system used for the electrochemical performance tests in this paper is a three-electrode system, consisting of a working electrode, a counter electrode, and a reference electrode. The working electrode is made of active material loaded on nickel foam collector, the counter electrode is platinum electrode, the reference electrode is Hg/HgO, and the electrolyte in the test environment is 6 M KOH solution. The equipment used was obtained from the Shanghai Chenhua Instrument Company (CHI-660E), and the tests were cyclic voltammetry (CV), with a voltage window of 0–0.6 V; electrochemical impedance spectroscopy (EIS), with a frequency of 0.01–100,000 Hz; and constant-current charge/discharge (GCD), with a voltage window of 0–0.5 V.

## 3. Results and Discussion

### 3.1. FT-IR Results

**Figure 2:**
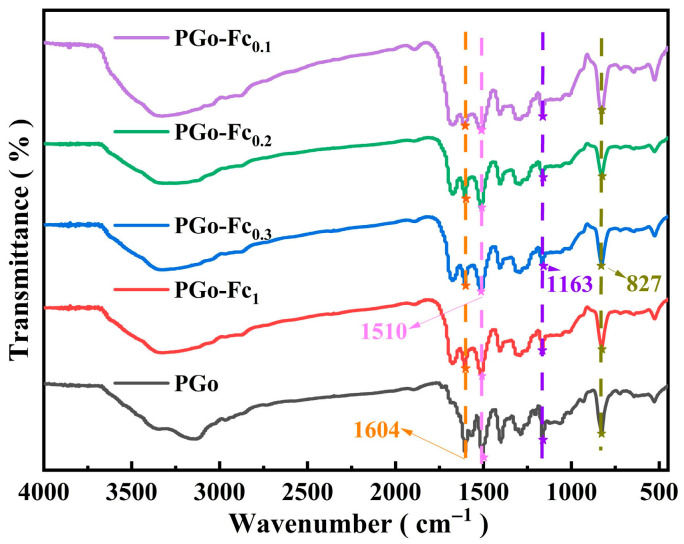
FT-IR spectra of PGo and PGo-Fc_x_.

FT-IR can qualitatively analyze the functional groups and chemical bonds of a material, which shows its different characteristic absorption peaks. From Figure 2, it can be found that the absorption peak seen at 1604 cm^−1^ belongs to the C=N bond, while a group of absorption peaks at 1570 cm^−1^, 1506 cm^−1^, and 1400 cm^−1^ can be attributed to the C=C stretching vibration in the benzene ring skeleton. The absorption peak located at 1163 cm^−1^ was caused by the in-plane stretching vibration and that at 828 cm^−1^ was the substitution of the benzene ring in the para position. The appearance of the above characteristic peaks can prove the successful preparation of a poly-Schiff base. For the ferrocene-doped PGo-Fc_x_ composites, the FT-IR spectra were similar to those of PGo, which indicated that the doping of ferrocene did not affect the PGo’s structure. The characteristic peaks of the PGo-Fc_x_ changed from 1604 cm^−1^ to 1606 cm^−1^. The absorption peak of the PGo-Fc_x_ at 1510 cm^−1^ was caused by the C=C stretching vibration of the benzene ring, the absorption peak near 1163 cm^−1^ was due to the C-N stretching vibration, and the absorption peak at 827 cm^−1^ came from the para-substitution of the benzene ring [21].

### 3.2. XRD Results

**Figure 3:**
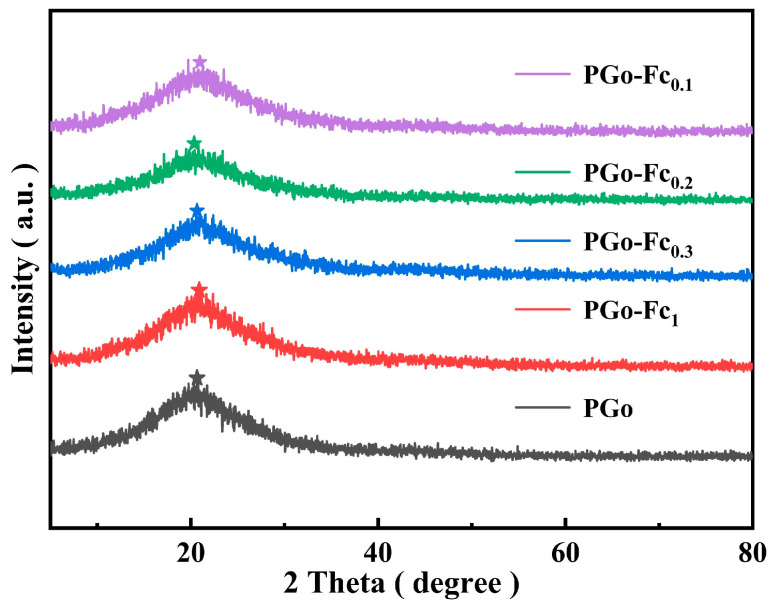
XRD spectra of PGo and PGo-Fc_x_.

The XRD spectra of the PGo-Fc_x_ electrode material are shown in Figure 3. The diffraction peak of the PGo was located at 20.6°, while the diffraction peak of PGo-Fc_x_ moved to the vicinity of 21° [20]. The XRD spectra of the PGo and PGo-Fc_x_ were similar, with a slight difference in the angle of the diffraction peak, which indicated that the ferrocene doping had a certain effect on PGo but that it did not cause any change in the crystalline state of the PGo. The doping of ferrocene made the diffraction peaks wider, which indicated that doping can make the grain size smaller [22].

### 3.3. SEM Results

**Figure 4:**
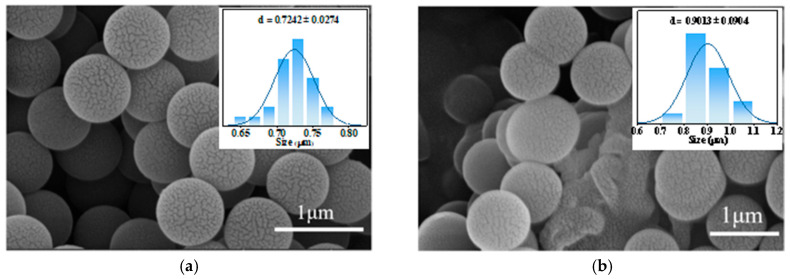

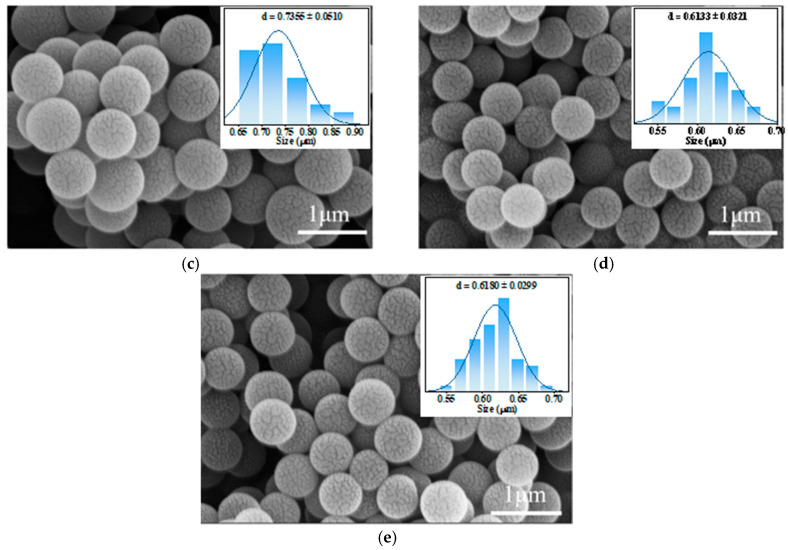
SEM images of PGo-Fc_x_ with different doping amounts. (**a**) PGo. (**b**) PGo-Fc_1_. (**c**) PGo-Fc_0.3_. (**d**) PGo-Fc_0.2_. (**e**) PGo-Fc_0.1_.

A scanning electron microscope model SU8000 manufactured by Hitachi, Japan was used for testing. The SEM images reflect the effect of different ferrocene doping amounts on the morphology of the PGo. The obtained microscopic morphology images of PGo and PGo-Fc_x_ electrode materials are shown in Figure 4. From Figure 4a, it can be observed that the PGo had a nano-spherical structure with some tiny cracks distributed on its surface, which was conducive to the full contact between the electrolyte and electrode materials and thus increased the capacitance performance. Figure 4b–e shows the SEM images of PGo-Fc_1_, PGo-Fc_0.3_, PGo-Fc_0.2_, and PGo-Fc_0.1_, respectively. The PGo-Fc_x_ material had a nanorodular structure, similarly to the PGo, and there were tiny cracks on the surface of PGo-Fc_0.3_, which also promoted the contact between the electrolyte and the active material. The difference between the PGo-Fc_x_ and PGo was that the particle size of PGo-Fc_1_ was smaller compared with that of PGo, whereas the particle sizes of PGo-Fc_0.3_, PGo-Fc_0.2_, and PGo-Fc_0.1_ were significantly smaller than those of PGo and PGo-Fc_1_, which was mainly due to the π–π conjugation of poly-Schiff bases by the dosage of the appropriate amount of ferrocene, which increased the specific surface area and reduced the volume. The specific surface area increasing was also beneficial in increasing the contact area between the electrolyte and the active material [21,23]. Therefore, the specific capacitances of PGo-Fc_0.3_, PGo-Fc_0.2_, and PGo-Fc_0.1_ were much higher than those of PGo and PGo-Fc_1_. Table 2 shows the average grain size of the PGo and PGo-Fc_x_.

**Figure 5:**
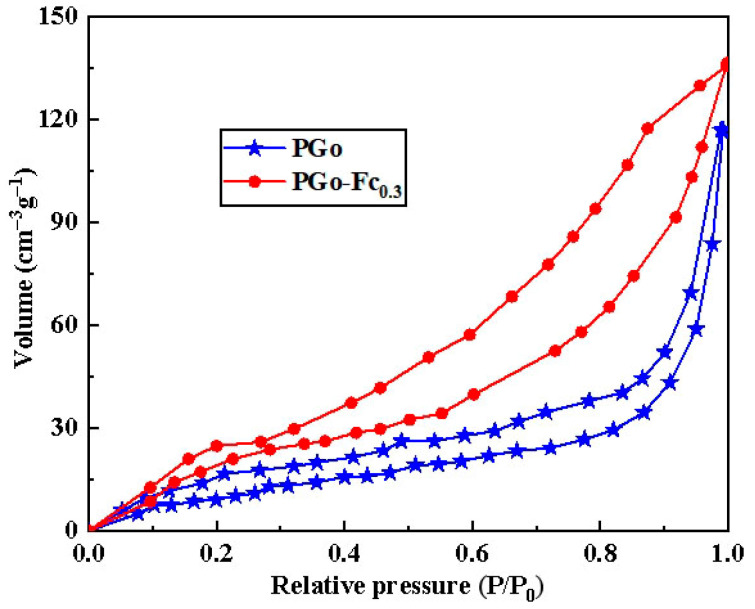
The N_2_ adsorption–desorption isotherm of PGo and PGo-Fc_0.3_.

Figure 5 shows the N_2_ adsorption–desorption isotherm plot, which illustrates the variation in adsorption volume as a function of the relative pressure (P/P_0_) for two materials: PGo(blue star lines) and PGo-Fc0.3(red dot lines). The two curves in the figure show the adsorption behavior of the two materials under increasing relative pressure. As the relative pressure (P/P_0_) increased from 0 to 1, the adsorption volume of both the PGo and PGo-Fc_0.3_ increased gradually. This was a typical characteristic of the adsorption isotherms, and indicating that more gas molecules are adsorbed onto the material surfaces as the pressure increases. The differences in the adsorption isotherms between the PGo and PGo-Fc_0.3_ suggests that the modification of the PGo with Fc_0.3_ was an effective way to improve its gas adsorption capacity. This finding had potential applications in areas such as gas storage, separation, and sensing, where materials with a high adsorption capacity are highly desirable. Table 3 presents the BET results for the PGo and PGo-Fc_0.3_. The differences in specific surface area, total pore volume, and average pore size between the two materials highlighted the impact of the Fc_0.3_ modification on the physical structure of the PGo.

### 3.4. Electrochemical Characterization of PGo-Fc_x_

CV, EIS, and GCD was undertaken to investigate the electrochemical properties of PGo-Fc_x_. The tests were carried with a three-electrode system using a homemade collector as the working electrode, with the amount of active substance being between 1 mg and 2 mg. The detailed working electrode preparation process was described in the Section 2.2. The counter electrode was a platinum electrode and the reference electrode was a Hg/HgO electrode with 6 M KOH solution electrolyte. The voltage for the CV was in the range of 0–0.6 V and the scanning rates were 5 mV/s, 10 mV/s, 20 mV/s, 50 mV/s, and 100 mV/s. The EIS frequency was in the range of 0.01–100,000 Hz, with the amplitude of the wave being 5 mV. The voltage for the GCD was in the range of 0–0.5 V and the current densities of the tests were 0.5 A/g, 1 A/g, 3 A/g, 5 A/g, and 10 A/g, respectively.

#### 3.4.1. CV Results

**Figure 6:**
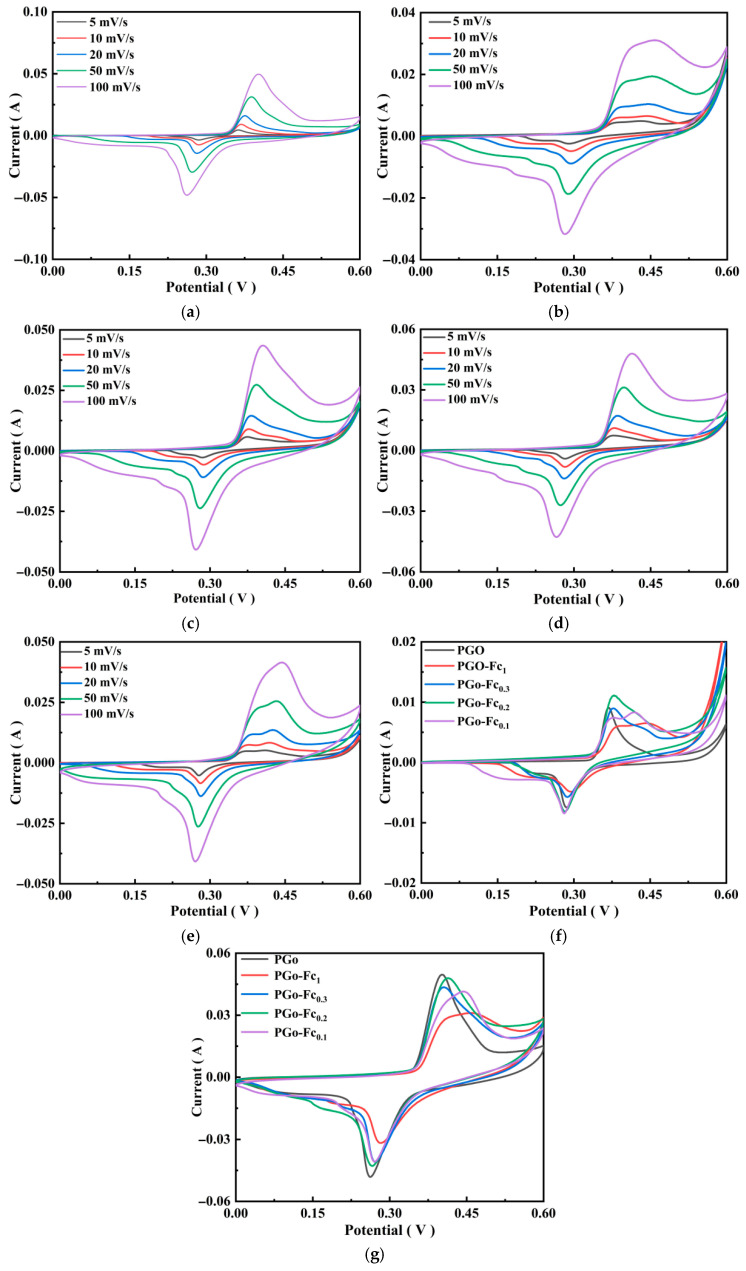
CV plots of PGo, PGo-Fc_1_, PGo-Fc_0.3_, PGo-Fc_0.2_, and PGo-Fc_0.1_ at different scanning rates. (**a**) PGo. (**b**) PGo-Fc_1_. (**c**) PGo-Fc_0.3_. (**d**) PGo-Fc_0.2_. (**e**) PGo-Fc_0.1_. (**f**) 10 mV/s. (**g**) 100 mV/s.

The CV results reflect the energy storage mechanism and electrochemical performance of the electrode material. The larger the integrated area of the CV curve, the better the electrochemical performance. As shown in Figure 6a–e, there were a pair of redox peaks in PGo, PGo-Fc_1_, PGo-Fc_0.3_, PGo-Fc_0.2_, and PGo-Fc_0.1_, which indicated that they are the type of pseudocapacitive electrode material that stores charges through redox reactions [24]. At the same time, the oxidation peaks and reduction peaks had good symmetry, which indicated that the electrode materials have good reversibility. The oxidation peaks and reduction peaks in the CV curves shifted to higher and lower potentials as the scanning rate increased from 5 mV/s to 100 mV/s, respectively, which was due to the polarization under high current density. The CV curves of PGo, PGo-Fc_1_, PGo-Fc_0.3_, PGo-Fc_0.2_, and PGo-Fc_0.1_ maintained good stability at high scanning rates of 10 mV/s and 100 mV/s, which indicated that the doped PGo still had good rate performance. From Figure 6g,f, it can be seen that PGo-Fc_0.3_ and PGo-Fc_0.2_ had larger CV integration areas than those of PGo-Fc_1_ and PGo-Fc_0.1_ at the 10 mV/s and 100 mV/s scanning rates, which suggested that PGo-Fc_0.3_ and PGo-Fc_0.2_ have a better capacitance performance.

#### 3.4.2. GCD Results

The GCD results indicate the character and specific capacitance performance of the electrode materials. The specific capacitance values of PGo, PGo-Fc_1_, PGo-Fc_0.3_, PGo-Fc_0.2_, and PGo-Fc_0.1_ at different current densities are shown in Table 4. The specific capacitance was tested by the three-electrode system, which was given by the formulation:

(1) $$C_{p}=\frac{I\cdot ∆t}{m\cdot ∆V}$$

where C_p_ is the specific capacitance, F/g; I is the current, A; m is the active substance mass, g; Δt is the discharging time, s; ΔV is the voltage, V.

**Figure 7:**
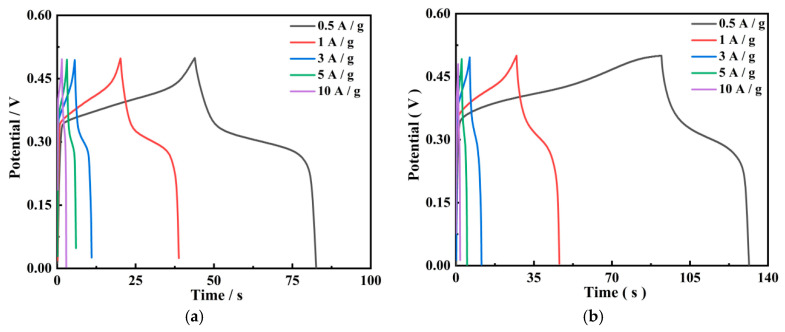

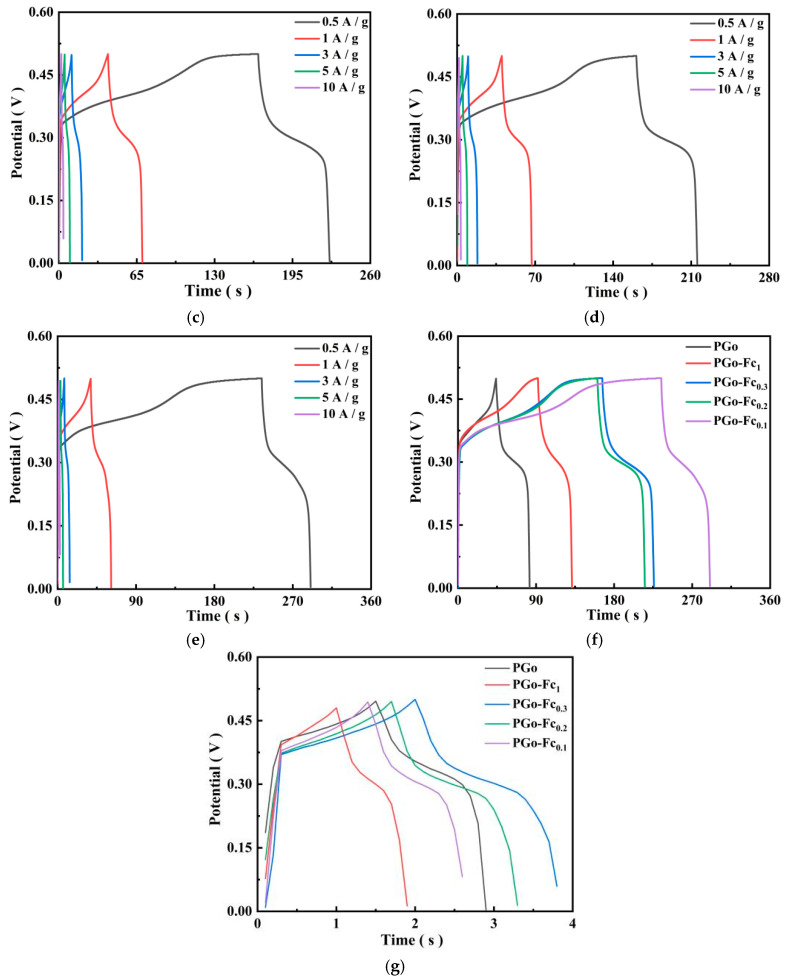
GCD plots of PGo, PGo-Fc_1_, PGo-Fc_0.3_, PGo-Fc_0.2_, and PGo-Fc_0.1_ at different current densities. (**a**) PGo. (**b**) PGo-Fc_1_. (**c**) PGo-Fc_0.3_. (**d**) PGo-Fc_0.2_. (**e**) PGo-Fc_0.1_. (**f**) 0.5 A/g. (**g**) 10 A/g.

The GCD results for PGo, PGo-Fc_1_, PGo-Fc_0.3_, PGo-Fc_0.2_, and PGo-Fc_0.1_ are shown in Figure 7a–e. The charging–discharging curves of PGo and PGo-Fc_x_ had a plateau and the charging–discharging curves of PGo had an approximate symmetry. The symmetry of PGo-Fc_x_ was poorer. The above research results still proved that PGo-Fc_1_, PGo-Fc_0.3_, PGo-Fc_0.2_, and PGo-Fc_0.1_ are pseudocapacitive materials.

From Figure 7f–g it can be seen that, compared with other PGo-Fc_x_ electrode materials, PGo-Fc_0.3_ had a longer discharging time, which indicates a better capacitance performance than PGo-Fc_x_ and PGo. PGo-Fc_0.3_ had a specific capacitance of 59.6 F/g at a current density of 0.5 A/g and 36.0 F/g at a current density of 10 A/g. The GCD plots of PGo and PGo-Fc_x_ did not undergo large distortion at a current density of 10 A/g, which indicates that they had good rate performance, which was consistent with the results of the CV. At a high current density, the reduction in discharging time was mainly due to the inability of the electrolyte ions to fully diffuse into the electrode material, which resulted in the decrease in total capacitance [25]. The specific capacitances of PGo, PGo-Fc_1_, PGo-Fc_0.3_, PGo-Fc_0.2_, and PGo-Fc_0.1_ were 36 F/g, 39.3 F/g, 54.7 F/g, and 49.1 F/g at a current density of 0.5 A/g, respectively.

#### 3.4.3. EIS Results

**Figure 8:**
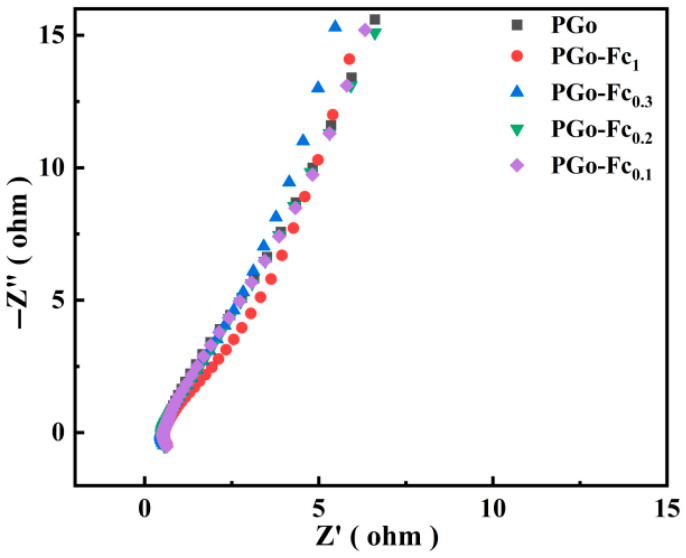
Nyquist plot of PGo, PGo-Fc_1_, PGo-Fc_0.3_, PGo-Fc_0.2_, and PGo-Fc_0.1_.

The Nyquist results indicated that the charge transfer resistance (Rct) can be determined by the semicircle diameter in the high-frequency region. The intersection of the semicircle and horizontal axis determined the solution resistance (Rs) and the slope in low-frequency region represents the Warburg impedance (W). Figure 8 shows the Nyquist plots of the PGo and PGo-Fc_x_ after the EIS testing. The Rs of the PGo, PGo-Fc_1_, PGo-Fc_0.3_, PGo-Fc_0.2_, and PGo-Fc_0.1_ were 0.53 Ω, 0.57 Ω, 0.47 Ω, 0.49 Ω, and 0.54 Ω. Both the PGo and PGo-Fc_x_ had very low solution resistance. The W in the low-frequency range was related to the linear slope, which responded to the diffusion rate between the electrode material and the electrolyte interface [26]. PGo-Fc_0.3_ had the largest slope, which suggests that the electrolyte ions had a faster diffusion rate between the PGo-Fc_0.3_ electrode and the electrolyte. Combining the Rs and W, we determined that the PGo-Fc_0.3_ electrode can more efficiently accelerate the electrode reaction kinetic process than the other electrodes, which was beneficial for the electrode charges storage and electron transferring.

#### 3.4.4. Rate and Cycle Performance

**Figure 9:**
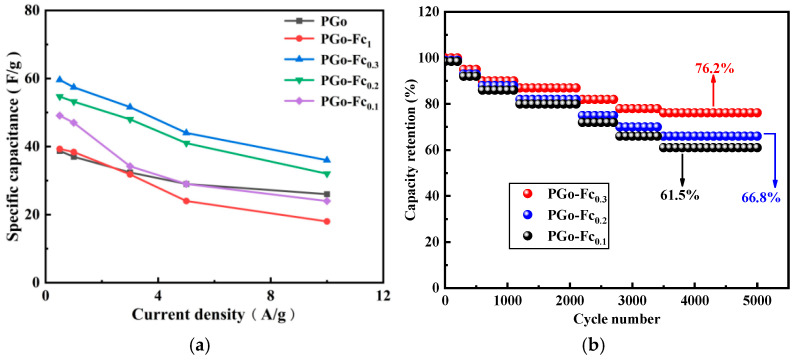
Performance of PGo, PGo-Fc_1_, PGo-Fc_0.3_, PGo-Fc_0.2_, and PGo-Fc_0.1_. (**a**) Rate performance. (**b**) Cycle performance.

Figure 9a shows the rate performance of the PGo, PGo-Fc_1_, PGo-Fc_0.3_, PGo-Fc_0.2_, and PGo-Fc_0.1_ at current densities ranging from 0.5 A/g to 10 A/g. PGo-Fc_0.3_ and PGo-Fc_0.2_ had better capacitance performance, while their rate performance was more stable. The decay trend of their specific capacitance was similar. Compared with PGo-Fc_0.2_, PGo-Fc_0.3_ had the higher specific capacitance at current densities ranging from 0.5 A/g to 10 A/g, while the rate performance of PGo-Fc_0.3_ was the highest among PGo-Fc_1_, PGo-Fc_0.3_, PGo-Fc_0.2_, and PGo-Fc_0.1_. The specific capacitance of 59.6 F/g was 0.5 A/g and that of 36 F/g was 10 A/g, whereas the rate performances of PGo-Fc_1_, PGo-Fc_0.2_, and PGo-Fc_0.1_ were 45.8%, 58.5%, and 48.9%, respectively. In order to evaluate its cycle stability, PGo-Fc_0.3_ was subjected to 5000 cycles of charging and discharging at a current density of 10 A/g. Figure 9b shows the cycle stabilities of PGo-Fc_0.3_, PGo-Fc_0.2_, PGo-Fc_0.1_ after 5000 cycles. It can be seen that PGo-Fc_0.3_ still maintained 76.2% capacitance, demonstrating a good cycle stability.

#### 3.4.5. Charge Storage Mechanism

**Figure 10:**
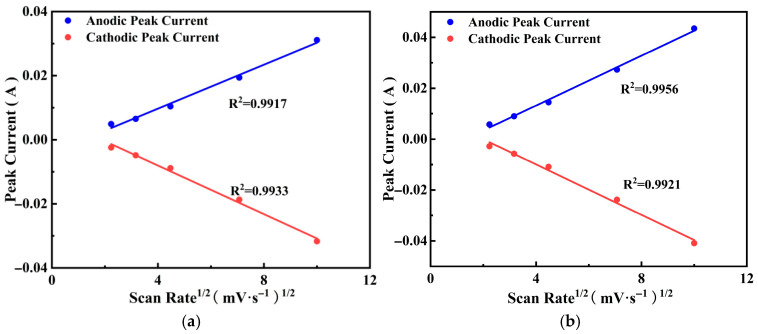

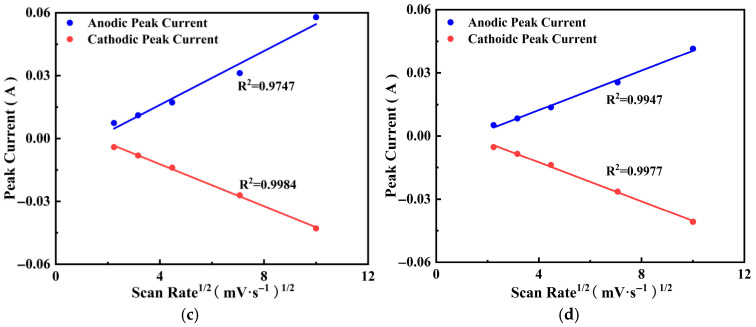
Linear fitting plots of the arithmetic square root mV^1/2^ for peak current and sweep speed of PGo-Fc_x_. (**a**) PGo-Fc_1_. (**b**) PGo-Fc_0.3_. (**c**) PGo-Fc_0.2_. (**d**) PGo-Fc_0.1_.

The linear fitting plots of the peak current *i* and the arithmetic squared root mV_1/2_ for PGo-Fc_1_, PGo-Fc_0.3_, PGo-Fc_0.2_, and PGo-Fc_0.1_ are shown in Figure 10a–d. The relationship was shown using the following equation.

The above three-electrode system has the following formulation:

(2) $$i_{p}=a\cdot v^{b}$$

where i_p_ is the peak current, A; v is the potential scan rate, mV/s; a and b are parameters. Taking the logarithm of Equation (2), we obtain the following:

(3) $$\log i_{p}=\log a+b\log v$$

If the b value was 0.5, it represented that the Faraday process dominated by diffusion and, if the b value was 1.0, it represented surface capacitance behavior. From Figure 10a–d, the good linear fitting relationship between the arithmetic squared root of the peak current and the scanning rate for the PGo-Fc_1_, PGo-Fc_0.3_, PGo-Fc_0.2_, and PGo-Fc_0.1_ electrode materials can be seen.

**Figure 11:**
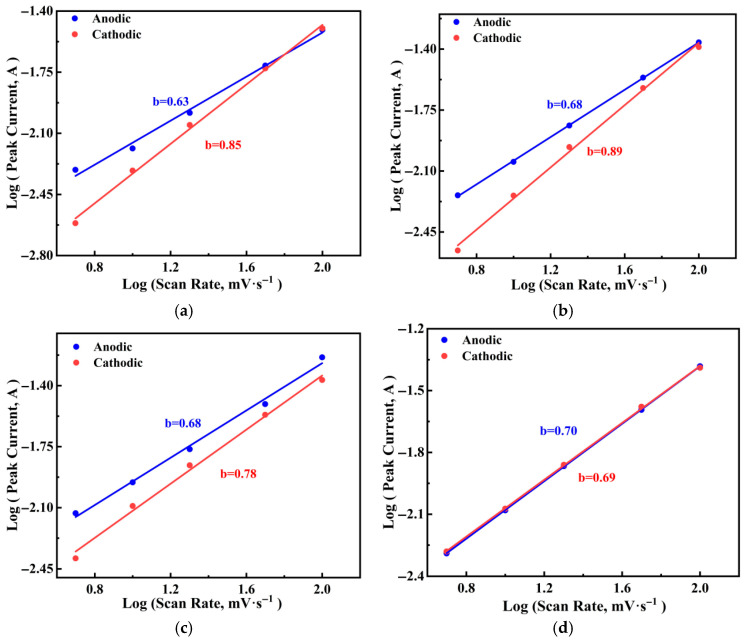
The relationship between the log peak current and the log scanning rate of PGo-Fc_x_. (**a**) PGo-Fc_1_. (**b**) PGo-Fc_0.3_. (**c**) PGo-Fc_0.2_. (**d**) PGo-Fc_0.1_.

The relationship between the log function log (*ip*) of the peak current and the log function log (*v*) of the scanning rate for PGo-Fc_1_, PGo-Fc_0.3_, PGo-Fc_0.2_, and PGo-Fc_0.1_ is shown in Figure 11a–d, and the calculation formulation is shown in Equation (3). It can be seen that the capacitance was mainly controlled by the diffusion when the b value was 0.5 and that the capacitance was mainly controlled by the surface capacitance when the b value was 1. The b values of PGo-Fc_1_ were 0.63 and 0.85, they were 0.68 and 0.89 for PGo-Fc_0.3_, they were 0.68 and 0.78 for PGo-Fc_0.2_, and they were 0.70 and 0.69 for PGo-Fc_0.1_, all of which are in the range of 0.5–1. Combined with the GCD and CV results in the previous section, PGo-Fc_1_, PGo-Fc_0.3_, PGo-Fc_0.2_, and PGo-Fc_0.1_ were determined to be pseudocapacitor-type electrode materials, which is the same type as that of PGo.

**Figure 12:**
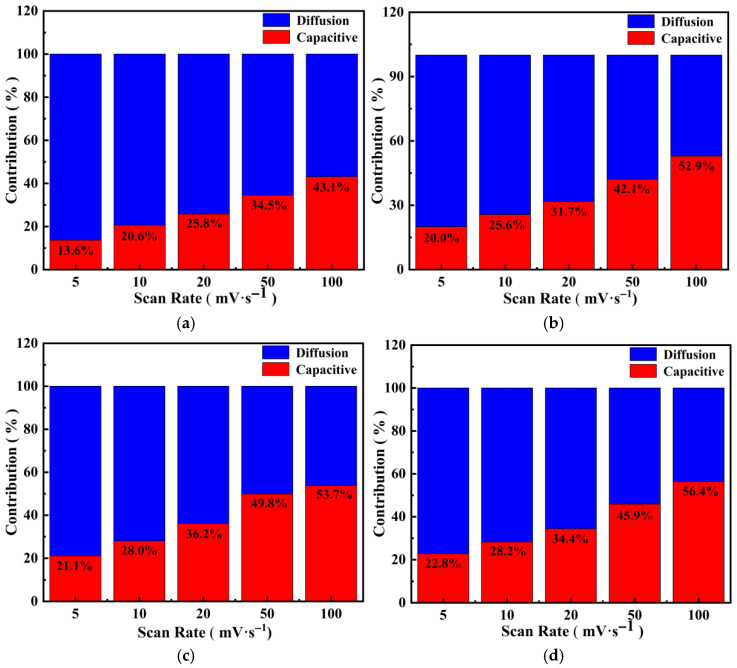
Contribution of PGo-Fc_x_ diffusion control (blue) and capacitance control (red) to the total capacitance at different scanning rates. (**a**) PGo-Fc_1_. (**b**) PGo-Fc_0.3_. (**c**) PGo-Fc_0.2_. (**d**) PGo-Fc_0.1_.

The normalized contributions of PGo-Fc_1_, PGo-Fc_0.3_, PGo-Fc_0.2_, and PGo-Fc_0.1_ to the diffusion control and surface capacitance behavior at different scanning rates are shown in Figure 12a–d. The capacitance was mainly controlled by the diffusion when PGo-Fc_0.3_ was charging and discharging at 5 mV/s, and with the increasing scanning rate, the contribution of the surface capacitance increased. When the scanning rate reached 100 mV/s, the surface capacitance contribution increased and reached52.9%, which was mainly contributed to by the surface capacitance. At a high scanning rate, rapid electrochemical reaction prevented ions in the electrolyte from completely diffusing into the electrode material, which resulted in a decrease in the total capacitance.

**Figure 13:**
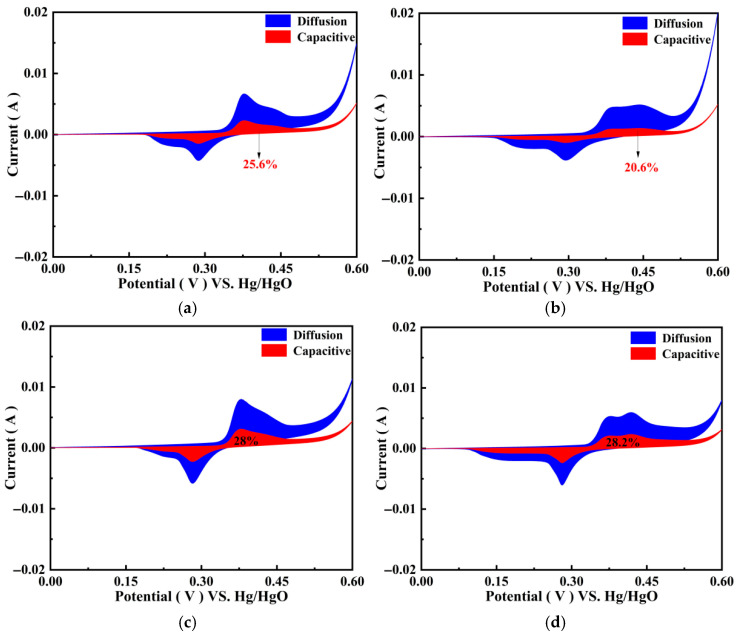
Contribution of PGo-Fc_x_ to diffusion control (blue) and surface capacitance (red). (**a**) PGo-Fc_1_. (**b**) PGo-Fc_0.3_. (**c**) PGo-Fc_0.2_. (**d**) PGo-Fc_0.1_.

Figure 13 shows a comparison between the diffusion control and surface capacitance control of PGo-Fc_1_, PGo-Fc_0.3_, PGo-Fc_0.2_, and PGo-Fc_0.1_ at a scanning rate of 10 mV/s. The red color represents the surface capacitance behavior and the blue color represented the diffusion control, which can be observed from Figure 13. The surface capacitance contribution of PGo-Fc_0.3_ was 25.6% at 10 mV/s, which indicated that the electrode material was mainly controlled by diffusion. The surface capacitance contributions of PGo-Fc_1_, PGo-Fc_0.2_, and PGo-Fc_0.1_ were 20.6%, 28.6%, and 28.2%, respectively, which are similar to that of PGo-Fc_0.3_. The same results as shown in Figure 12, which further demonstrates that the pseudocapacitive electrode materials of PGo-Fc_x_ and the charging storage mechanism of diffusion and surface capacitance contribute to the total capacitance together.

The surface capacitance contribution to the total capacitance continued to increase, and the PGo-Fc_x_ electrode materials exhibited similar properties. Therefore, the charge storage mechanism of the PGo-Fc_x_ material was mainly determined by diffusion-controlled and surface-controlled pseudocapacitive behavior [27].

## 4. Conclusions

PGo-Fc_x_ with a nanospherical structure was successfully prepared by one single step. According to electrochemical testing, PGo-Fc_0.3_ exhibited the best capacitance performance, with a rate performance of 60.4% and a specific capacitance of 59.6 F/g at 0.5 A/g and 36 F/g at 10 A/g. Due to PGo-Fc_x_ being a pseudocapacitive electrode material, the charging storage was determined by the pseudocapacitive behavior and the diffusion-controlled behavior.

## Figures and Tables

**Table 1:** The materials used in this paper.

Name of Material	Molecular Formula/Abbreviation	Norm
Toluene diamine	C_6_H_8_N_2_	Analytically pure (AR)
Glyoxal solution	C_2_H_2_O_2_	Analytically pure (AR)
Ferrocene	FeC_10_H_10_	Analytically pure (AR)
Anhydrous ethanol	C_2_H_6_O	Analytically pure (AR)
Deionized water	/	/
Potassium hydroxide	KOH	Analytically pure (AR)
Hydrochloric acid	HCl	Analytically pure (AR)

**Table 2:** The average grain size of PGo and PGo-Fc_x_.

	PGo	PGo-Fc_1_	PGo-Fc_0.3_	PGo-Fc_0.2_	PGo-Fc_0.1_
Average grain size	0.72 μm	0.90 μm	0.62 μm	0.73 μm	0.61 μm

**Table 3:** The BET results of PGo and PGo-Fc_0.3_.

Material	Specific Surface Area (m^2^/g)	Total Pore Volume (cm^3^/g)	Average Pore Size (nm)
PGo	36.73	0.086	24.5
PGo-Fc_0.3_	41.65	0.108	22.8

**Table 4:** The specific capacitance of PGo, PGo-Fc_x_, PB, and PGd at different current densities.

Electrode Material	Different Current Density				
	0.5 A/g	1 A/g	3 A/g	5 A/g	10 A/g
PGo	38.7 F/g	37.0 F/g	32.4 F/g	29.0 F/g	26.0 F/g
PGo-Fc_1_	39.3 F/g	38.4 F/g	31.8 F/g	24.0 F/g	18.0 F/g
PGo-Fc_0.3_	59.6 F/g	57.4 F/g	51.6 F/g	44.0 F/g	36.0 F/g
PGo-Fc_0.2_	54.7 F/g	53.2 F/g	48 F/g	41.0 F/g	32.0 F/g
PGo-Fc_0.1_	49.1 F/g	47.0 F/g	34.2 F/g	29.0 F/g	24.0 F/g
PB	21.7 F/g	20.8 F/g	20.4 F/g	20.0 F/g	18.0 F/g
PGd	16.3 F/g	15.6 F/g	14.4 F/g	14.0 F/g	12.0 F/g

## Data Availability

All data generated or analyzed during this study are included in this published article.
